# Human growth hormone and human prolactin function as autocrine/paracrine promoters of progression of hepatocellular carcinoma

**DOI:** 10.18632/oncotarget.8781

**Published:** 2016-04-18

**Authors:** Xiangjun Kong, Wenyong Wu, Yan Yuan, Vijay Pandey, Zhengsheng Wu, Xuefei Lu, Weijie Zhang, Yijun Chen, Mingming Wu, Min Zhang, Gaopeng Li, Sheng Tan, Pengxu Qian, Jo K. Perry, Peter E. Lobie, Tao Zhu

**Affiliations:** ^1^ The CAS Key Laboratory of Innate Immunity and Chronic Disease, School of Life Sciences and Medical Center, University of Science and Technology of China, Hefei, Anhui, China; ^2^ Hefei National Laboratory for Physical Sciences at Microscale, Hefei, Anhui, China; ^3^ Department of General Surgery, First Affiliated Hospital of Anhui Medical University, Hefei, Anhui, China; ^4^ Cancer Science Institute of Singapore and Department of Pharmacology, National University of Singapore, Singapore; ^5^ Department of Pathology, Anhui Medical University, Hefei, Anhui, China; ^6^ Liggins Institute, University of Auckland, Auckland, New Zealand; ^7^ National University Cancer Institute of Singapore, National University Health System, Singapore

**Keywords:** growth hormone, prolactin, hepatocellular carcinoma, oncogenicity, survival

## Abstract

The death rates of hepatocellular carcinoma (HCC) are extremely high due to the paucity of therapeutic options. Animal models and anecdotal clinical evidence indicate a potential role of hGH and hPRL in HCC. However, the prognostic relevance and the functional role of tumor expression of these hormones in human HCC are not defined. Herein, we analyzed the mRNA and protein expression of hGH and hPRL in histopathological samples of non-neoplastic liver and HCC by *in situ* hybridization, PCR and immunohistochemistry techniques. Increased mRNA and protein expression of both hormones was observed in HCC compared with non-neoplastic liver tissues. hGH expression was significantly associated with tumor size and tumor grade. No significant association was observed between the expression of hPRL and any histopathological features. Amplification of both hGH and hPRL genes in HCC was observed when compared to non-neoplastic tissue. Expression of both hGH and hPRL was associated with worse relapse-free and overall survival in HCC patients. *In vitro* and *in vivo* functional assays performed with HCC cell lines demonstrated that autocrine expression of hGH or hPRL in HCC cells increased STAT3 activation, oncogenicity and tumor growth while functional antagonism with hGH-G120R significantly reduced these parameters. Hence, tumor expression of hGH/hPRL is associated with a worse survival outcome for patients with HCC and hGH/hPRL function as autocrine/paracrine promoters of HCC progression.

## INTRODUCTION

In addition to their classic endocrine actions, human growth hormone (hGH) and human prolactin (hPRL) have been reported to function as autocrine and/or paracrine growth factors in tissues such as the mammary gland, endometrium, prostate and central nervous system including the retina [1]. Previous studies have also demonstrated that autocrine expression of hGH and hPRL promoted oncogenicity and progression of carcinomas derived from a range of tissues [2–6] and autocrine hGH may serve as a transforming oncogene at least for mammary epithelial cells [7]. Furthermore, independent of serum hGH, hPRL or IGF1 levels, expression of hGH or hPRL in mammary or endometrial carcinoma is associated with unfavorable histopathological features with a significantly worse survival outcome for patients [8]. Thus, both hGH and hPRL exert tissue and disease specific functions in an autocrine/paracrine manner.

Both the GH receptor and PRL receptor were first identified and characterized in liver [9, 10]. Indeed, the liver has been considered a predominant target organ for both GH and PRL [11]. In the human, hGH activates both the hGH receptor and the hPRL receptor [12]. The majority of GH dependent serum IGF1 is hepatic derived [13] and patients with hepatic cirrhosis exhibit decreased serum IGF1 with concomitant elevated GH [14] indicative of hGH resistance. Aberrations in the somatotropic axis have previously been implicated in the development of HCC. For example, mice transgenic for GH spontaneously develop HCC [15] and display enhanced carcinogen induced HCC [16]. In contrast, GH deficient mice are dramatically resistant to the development of carcinogen induced liver cancer [17]. Furthermore, hGH administration promotes growth of hGHR positive human hepatocellular and gastric carcinoma cell lines [18, 19]. Similar to GH, PRL has been reported to function as a tumor promoter for chemically initiated rat liver cells [20]. Recently, several investigations identified that serum PRL levels were significant elevated in HCC patients and PRL was one of the potential tumor markers for HCC, suggesting that hPRL may be useful as a biomarker for early detection of HCC and may play a role in HCC progression [21–23].

Although increased cellular expression of both the hGHR and hPRLR has been observed in HCC [24] and increased serum hGH and hPRL levels have been observed in HCC patients [25, 26], the potential tumor expression of hGH and hPRL, clinicopathological associations and prognostic significance remain unknown. Herein, we report the expression of hGH and hPRL in HCC, an association of hGH and hPRL expression with poor survival outcome and provide detailed *in vitro* and *in vivo* functional analyses which support an autocrine and/or paracrine role for both hGH and hPRL in human HCC progression.

## RESULTS

### Expression of hGH and hPRL in hepatocellular carcinoma and adjacent non-tumor tissue

**Figure 1:**
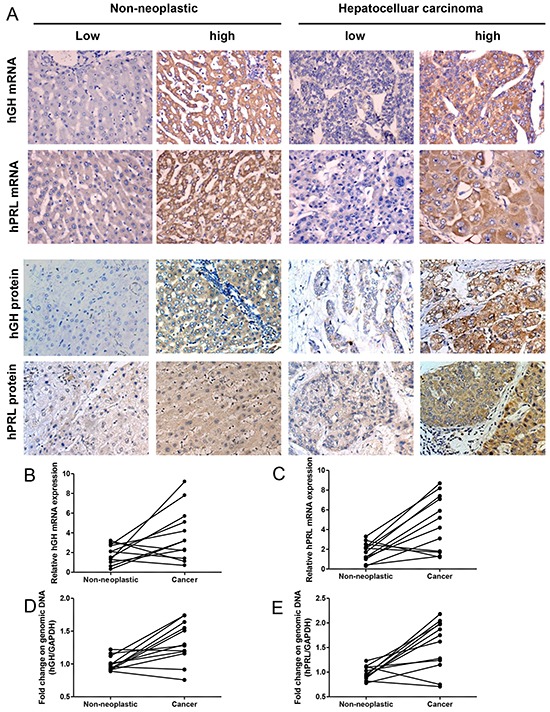
hGH or hPRL expression in hepatic non-neoplastic tissue and hepatocellular carcinoma **A.** ISH and IHC analysis of hGH and hPRL expression in non-neoplastic hepatic tissue and HCC. *Left two panels*, Expression of hGH and hPRL (mRNA and protein) in non-neoplastic hepatic tissue. *Right two panels*, Expression of hGH and hPRL (mRNA and protein) in HCC. Micrographs were captured at ×200 magnification. **B.** hGH mRNA levels in HCC and adjacent non-neoplastic tissues detected by qPCR. **C.** hPRL mRNA levels in HCC and adjacent non-neoplastic tissues detected by qPCR. qPCR on genomic DNA to detect hGH- **D.** and PRL- **E.** amplification in HCC and adjacent non-neoplastic tissues.

We utilized ISH to detect hGH or hPRL mRNA in HCC specimens (Figure 1A). Increased expression of both hGH and hPRL mRNA was observed in HCC specimens when compared with the corresponding adjacent non-neoplastic hepatic tissue (Supplementary Table S1).

We also determined the mRNA levels of hGH or hPRL in paired non-neoplastic hepatic tissue and HCC by qPCR. Concordant with the ISH results, eight of twelve patients showed increased expression of hGH mRNA in tumors compared to non-neoplastic hepatic tissue (Figure 1B). The mRNA levels of hPRL exhibited a similar pattern, nine of twelve patients showed increased expression of hPRL mRNA in tumor compared to non-neoplastic hepatic tissue (Figure 1C). We next determined if the genomic loci of hGH and hPRL were amplified in HCC. We therefore performed qPCR on genomic DNA extracted from HCC and adjacent non-neoplastic tissue. Five of twelve patients showed hGH genomic gain (>1.5 fold increase) and six of twelve patients showed hPRL genomic gain in tumors (Figure 1D,E). Consistent with our results, the Cancer Genome Atlas project (TCGA) via cBioPortal [27, 28] reported in 206 samples, that 18 (9%) and 13 (6%) tumor samples harbored genomic amplification or mRNA upregulation for hGH or hPRL, respectively.

hGH and hPRL protein expression in the carcinoma and adjacent non-neoplastic tissue from HCC patients exhibited similar (Figure 1A) but not identical patterns as compared to the respective mRNAs. As previously observed in mammary and endometrial tissues [8], such discrepancies may result from the differential sensitivity of ISH vs. IHC. Furthermore, hGH and hPRL are secretory proteins, which may alter cellular retention and/or localization. As shown in Supplementary Table S1, increased expression of hGH protein was detected in HCC tissues compared with non-tumorous hepatic tissue. Similarly, the expression of hPRL protein was strongly and significantly increased in HCC compared to that in non-tumorous tissue (48.3% and 7.8%). hPRL protein expression in the non-neoplastic liver tissue was similar but not identical to that observed with the mRNA (Supplementary Table S1).

### Correlation between expression of hGH and hPRL, clinicopathological features of HCC and patient survival

As observed in Supplementary Table S2, high expression of hGH mRNA was positively associated with larger tumor size and higher histological grade. However, no significant association was observed between the high expression of hPRL protein and any clinicopathological features of HCC patients. Interestingly, a significant association between the tumor expression of hGH protein or hPRL mRNA and gender was observed.

**Figure 2:**
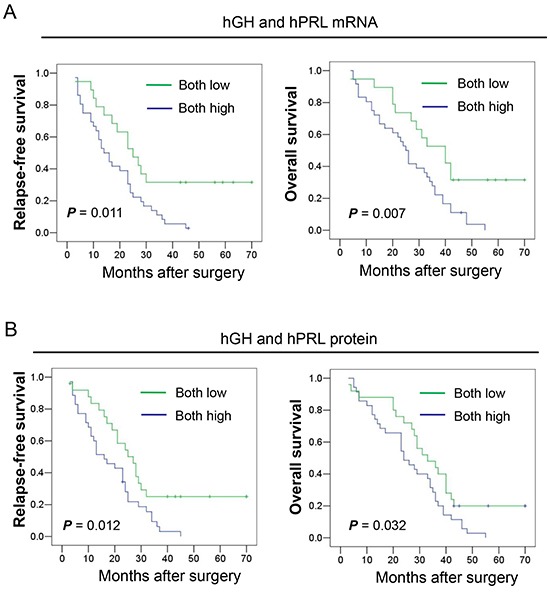
Kaplan-Meier analysis of the significance of hGH and hPRL expression on RFS and OS of patients with HCC **A.** The relationship of hGH and hPRL mRNA expression and RFS to OS of patients with HCC. **B.** The relationship of hGH and hPRL protein expression and RFS to OS of patients with HCC.

To determine whether hGH or hPRL expression in HCC is associated with RFS and OS, we performed Kaplan-Meier analyses on the cohort of patients with HCC. Patients whose tumors expressed low levels of hGH mRNA exhibited a mean 5 year RFS and OS rate of 31.3% and 28.1% respectively. In contrast, patients with tumors expressing a high level of hGH mRNA exhibited a mean 5 year RFS and OS rate of 8.8% and 5.9% respectively (Table 1). No significant correlation was observed between the expression of hGH protein and patient RFS or OS rate. Similarly, patients whose tumors express high levels of hPRL mRNA exhibited a significantly lower OS rate (but not RFS rate), compared to patients whose tumor exhibited low expression of hPRL mRNA. Moreover, patients whose tumors express a high level of hPRL protein exhibited a significantly lower RFS and OS compared to patients whose tumors expressed low levels of hPRL protein respectively. The RFS and OS of patients whose tumors exhibited low expression of both hGH and hPRL, either mRNA or protein, was higher than patients whose tumors exhibited high expression of either hGH or hPRL (Table 1, Figure 2A, 2B). Furthermore, the RFS and OS rates for patients whose tumors exhibited low expression of mRNA or protein for both hormones were significantly higher when compared to patients whose tumors were high expressing for both hGH and hPRL mRNA or protein expression.

**Table 1:** Association of tumor hGH or hPRL mRNA and hGH or hPRL protein expression with five year relapse free (RFS) and overall survival (OS) in patients with hepatocellular carcinoma

	Hepatocellular carcinoma							
	RFS (%)				OS (%)			
	mRNA	*P*	protein	*P*	mRNA	*P*	protein	*P*
hGH low/hGH high	31.3/8.8	**0.015**	22.0/11.9	0.191	28.1/5.9	**0.026**	17.1/10.2	0.286
hPRLlow/hPRL high	20.0/10.6	0.096	25.5/6.0	**0.009**	16.1/8.5	**0.044**	21.3/4.0	**0.016**
hGH low hPRL low /hGH high	31.6/7.6	**0.035**	28.0/10.7	**0.122**	31.6/4.5	**0.017**	20.0/8.9	0.254
hGH low hPRL low /hPRL high	31.6/10.6	0.067	28.0/6.0	**0.037**	31.6/8.5	**0.024**	20.0/4.0	0.111
hGH low hPRL low/hGH high hPRL high	31.6/2.8	**0.011**	28.0/2.9	**0.012**	31.6/2.8	**0.007**	20.0/0	**0.032**

Multivariate analyses also revealed that the adjusted odds ratios for death or relapse of patients with HCC were significantly increased in patients whose tumors expressed high levels of hGH or hPRL. The adjusted odds ratios are presented in Supplementary Table S3. Combined expression of both hGH and hPRL, at either the mRNA or protein level in HCC, was significantly associated with decreased RFS and OS.

We also examined whether a different prognostic significance of hGH and hPRL expression in HCC existed between genders. Interestingly, in the univariate Kaplan-Meier survival analyses, a significant association between the expression of hGH and hPRL and survival was only observed in males (Supplementary Table S4). No significant association was observed between the expression of such hormones and the survival of female patients. Furthermore, those males with the phenotype of hGH-high-hPRL-high exhibited a shorter OS and RFS than patients with any other phenotype of hGH or hPRL expression (Supplementary Figure S1).

### Expression of hGH, hPRL, hGHR and hPRLR mRNAs in HCC cell lines

**Figure 3:**
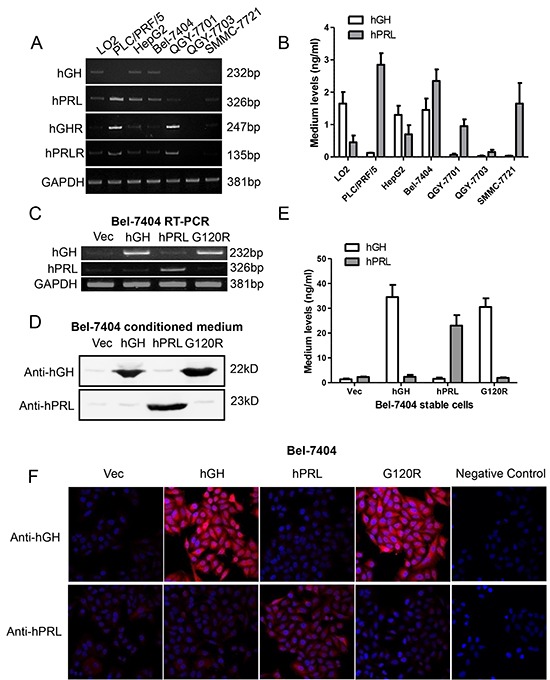
Forced expression of hGH, hPRL and G120R in Bel-7404 cells **A.** RT-PCR detection of mRNA expression of hGH, hPRL, hGHR and hPRLR in a panel of cell lines. **B.** ELISA detection of secreted hGH and hPRL **C.** RT-PCR analyses of forced expression of hGH, hPRL and G120R in Bel-7404 cells. Immunoblot **D.** ELISA **E.** and immunofluorescent **F.** analyses of forced expression and secretion of hGH, hPRL and G120R in Bel-7404 cells. Mean +/− SD.

We next examined hGH or hPRL mRNA expression in a number of HCC cell lines by RT-PCR. As observed in Figure 3A, hGH mRNA was expressed in HepG2, Bel-7404 and a normal immortalized human liver cell line LO2. hPRL mRNA was expressed in LO2 and almost all HCC cell lines except QGY-7703. hGHR and hPRLR mRNA expression patterns were similar and were expressed in all cell lines examined except QGY-7703. ELISA detection of secreted hGH and hPRL protein in cell medium demonstrated that hGH and hPRL levels vary from 0.02 and 0.1 to 1.9 and 2.6 ng/ml respectively (Figure 3B).

### Autocrine expression of hGH or hPRL promote HCC cell proliferation and survival

To determine whether autocrine expression of hGH or hPRL modulates HCC cell behavior, we stably transfected Bel-7404 and HepG2 cells with plasmids encoding hGH or hPRL cDNA or with the empty plasmid. To inhibit endogenously produced hGH and hPRL, we established another cell line in which cells were transfected with a plasmid that expresses hGH-G120R. G120R is a hGH analogue with a single amino acid substitution at position 120 which acts as a dual hGH and hPRL antagonist and antagonizes signaling from both receptors [29].

The forced expression and secretion of hGH, hPRL and G120R in Bel-7404 and HepG2 cells were verified by RT-PCR (Figure 3C and Supplementary Figure S2A), immunoblot (Figure 3D and Supplementary Figure S2B) and ELISA analysis (Figure 3E and Supplementary Figure S2C). Expression was also verified by immunofluorescence in Bel-7404 stable cell lines (Figure 3F).

**Figure 4:**
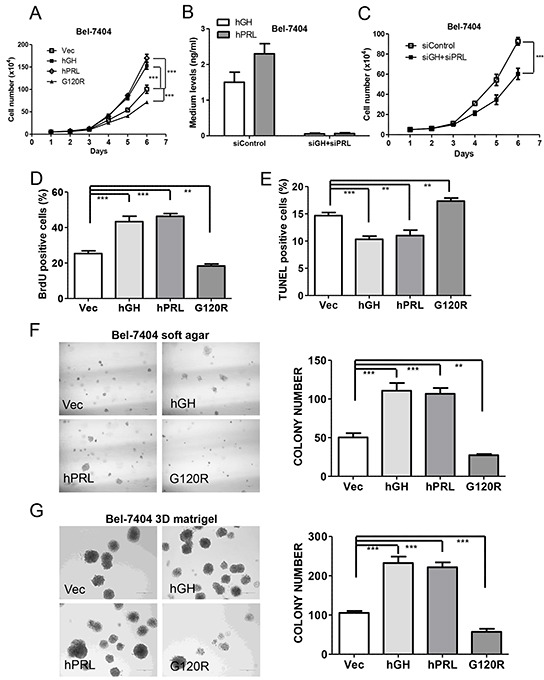
Autocrine expression of hGH or hPRL promote HCC cell oncogenicity *in vitro* **A.** Growth of Bel-7404 cell lines was assessed by a total cell number assay in complete medium. **B.** ELISA detection of hGH and hPRL levels in medium of Bel-7404 cells with tranfection of control siRNA or combined transfection of hGH and hPRL siRNAs. **C.** Growth of Bel-7404 cells with combined transfection of hGH and hPRL siRNAs by a total cell number assay in complete medium. Effect of autocrine expression of hGH, PRL or G120R on nuclear BrdU incorporation in complete medium **D.** on apoptosis induced by serum withdrawal over 48 h as evaluated by TUNEL assay **E.** on Soft agar colony formation **F.** and 3D Matrigel growth **G.** ** p<0.01, *** p<0.001. Mean +/− SD.

Forced expression of hGH or hPRL in Bel-7404 and HepG2 cells increased total cell number (Figure 4A and Supplementary Figure S3A). In contrast, the proliferation rate of both cell lines with forced expression of G120R decreased significantly (Figure 4A and Supplementary Figure S3A). We also utilized siRNAs to deplete both endogenous hGH and hPRL (Figure 4B and Supplementary Figure S3B). Total cell number assay demonstrated that the proliferation rate of cells with combined transfection of hGH and hPRL siRNAs were significantly decreased compared with control transfected cells (Figure 4C and Supplementary Figure S3C). Autocrine expression of hGH or hPRL also significantly increased Bel-7404 and HepG2 cell entry into S-phase as determined by BrdU incorporation (Figure 4D and Supplementary Figure S3D). Cell cycle progression was significantly decreased in both cell lines with forced expression of G120R (Figure 4D and Supplementary Figure S3D). Autocrine hGH or hPRL also decreased apoptotic cell death in serum deprived conditions whereas G120R promoted cell apoptosis (Figure 4E and Supplementary Figure S3E). Autocrine hGH or hPRL also significantly increased Bel-7404 and HepG2 cell colony formation in soft agar (Figure 4F and Supplementary Figure S3F) and 3-dimensional growth in Matrigel (Figure 4G and Supplementary Figure S3G) whereas cells with forced expression of G120R formed less colonies.

To further verify the paracrine effects of both hormones, we exposed parental HCC cells to the conditioned medium collected from cells with forced expression of hGH or hPRL. Total cell number assay demonstrated that the proliferation rate of both Bel-7404 and HepG2 cells cultured with the respective conditioned medium from cells with forced expression of hGH or hPRL was significantly increased compared with cells cultured with conditioned medium from the respective vector expressing cells. (Supplementary Figure S4A and S4B).

### Autocrine expression of hGH or hPRL modulate gene expression in Bel-7404 cell

Hepatic production of insulin like growth factors (IGFs) partially mediate the somatic effects of hGH [30]. We therefore ascertained whether autocrine expression of hGH or hPRL modulated IGF1 and IGF2 mRNA levels in Bel-7404 cells by qPCR. Both IGF1 and IGF2 mRNA levels were increased in Bel-7404-hGH cells when compared with control cells (Supplementary Figure S5A, 5B). Surprisingly, autocrine expression of hPRL predominantly promoted IGF1 mRNA expression whereas hGH predominantly stimulated IGF2 mRNA expression (Supplementary Figure S5A, 5B). Both IGF1 and IGF2 mRNA levels were decreased in Bel-7404 cells with forced expression of G120R (Supplementary Figure S5A, B). Furthermore, qRT-PCR analysis on Bel-7404 cell lines demonstrated altered expression of various genes associated with signal transduction, cell cycle progression, cell survival and inflammation by forced expression of hGH or hPRL (Supplementary Table S5). Forced expression of G120R, in general, produced opposing changes in gene regulation to those observed with hGH or hPRL (Supplementary Table S5).

### Autocrine expression of hGH or hPRL promote HCC xenograft growth *in vivo*

**Figure 5:**
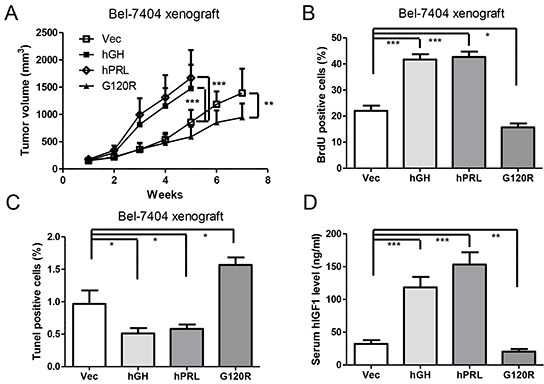
Autocrine expression of hGH or hPRL enhances HCC cell tumor growth *in vivo* **A.** Tumor volume in relation to the day of surgery is shown. **B.** Evaluation of nuclear BrdU incorporation in tumors. **C.** Evaluation of TUNEL positive (apoptotic) nuclei in tumors. **D.** Mouse serum human IGF1 levels were examined by ELISA. * p<0.05, ** p<0.01, *** p<0.001. Mean +/− SD.

To determine whether autocrine expression of hGH or hPRL enhances HCC growth *in vivo*, we implanted Bel-7404 stable cells subcutaneously in athymic nude mice. Bel-7404 cells with forced expression of hGH or hPRL formed markedly larger tumors and G120R expressing cells produced significantly smaller tumors (Figure 5A). Subsequent quantification of cell proliferation and apoptosis by BrdU or TUNEL labeling on tumor sections demonstrated that hGH or hPRL expressing tumors exhibited higher proliferation and lower apoptosis (Figure 5B, 5C). In contrast, tumors with expression of G120R exhibited less proliferation and higher rates of apoptosis (Figure 5B, 5C).

The levels of human IGF1 protein in mouse serum were observed to be significantly increased in hGH or hPRL expressing tumor bearing mice (Figure 5D). Serum hIGF1 levels of mice bearing tumors with G120R expressing were decreased compared with control mice (Figure 5D).

### STAT3 signaling is required for autocrine hGH or hPRL stimulated oncogenicity

**Figure 6:**
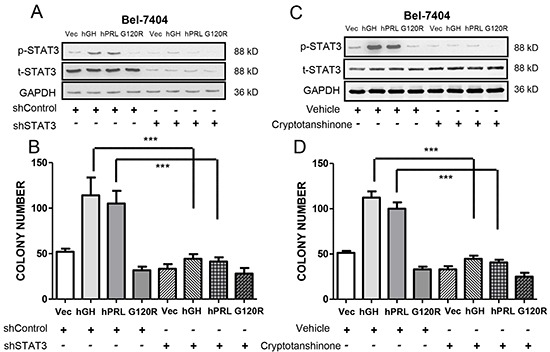
hGH and hPRL stimulated oncogenicity is mediated by STAT3 **A.** Immunoblot analysis of total STAT3 and pSTAT3 Y705 levels after STAT3 shRNA transfection. **B.** Soft agar colony formation of Bel-7404 cells transfected with STAT3 shRNA. **C.** Immunoblot analysis of total STAT3 and pSTAT3-Y705 levels after cryptotanshinone treatment (10 μM). **D.** Soft agar colony formation of Bel-7404 cells treated with cryptotanshinone. * p<0.05, ** p<0.01, *** p<0.001. Mean +/− SD.

GH has been reported to activate STAT5 in liver [31]. However, in HCC cells with forced expression of hGH or hPRL, we did not observe activation of STAT5 (as determined by phosphorylation of STAT5a/b on Tyr 694/Tyr 699) (data not shown). As hyperactivation of STAT3 has been postulated to be involved in HCC development [32] and both hGH and hPRL activate STAT3, we next ascertained whether STAT3 signaling was involved in hGH and hPRL stimulation of HCC oncogenicity. Levels of activated STAT3 (pSTAT3-Y705) were observed to be increased by autocrine expression of either hGH or hPRL and decreased by expression of G120R in Bel-7404 cells (Figure 6A). To determine if STAT3 signaling mediates hGH and hPRL stimulated oncogenicity, we depleted STAT3 in Bel-7404 cell lines by shRNA (Figure 6A). Increased colony formation stimulated by either hGH or hPRL in soft agar was significantly abrogated by STAT3 depletion (Figure 6B). Furthermore, treatment of cells with cryptotanshinone, a STAT3 specific inhibitor [33], which efficiently decreased pSTAT3-Y705 levels (Figure 6C), also resulted in inhibition of soft agar colony formation stimulated by autocrine expression of hGH or hPRL (Figure 6D). In addition, both hGH and hPRL would be expected to activate other signaling pathways in HCC cells and we observed activation of p44/42 MAP kinase (ERK1/2) in both cell lines with forced expression of hGH or hPRL. G120R correspondingly decreased p44/42 MAP kinase levels in HCC cells (Supplementary Figure S6).

## DISCUSSION

This study has demonstrated a significant association of tumor hGH expression with clinicopathological characteristics of HCC. Although we observed no significant association of tumor hPRL expression with any clinicopathological features of HCC, both hGH and hPRL expression were individually associated with poor survival of HCC patients overall and specifically in male HCC patients. Furthermore, and similar to that observed with both mammary and endometrial carcinoma [8], combined expression of hGH and hPRL predicted a worse survival outcome than that observed with either hormone individually. That tumor expression of hGH and hPRL promotes poor survival outcome in HCC is also consistent with functional assays in which autocrine expression of hGH or hPRL promoted oncogenicity of HCC cells. Again, similar oncogenic effects of autocrine expression of hGH have been demonstrated in mammary [7] and endometrial carcinoma cells [4] and for hPRL in mammary [34], endometrial and ovarian carcinoma cells [35].

IGF1 and IGF2 are expressed in human primary HCC cells and are involved in HCC development and progression [36]. It has been proposed that many of the effects of hGH on somatic growth are mediated through promotion of hepatic IGF1 synthesis and secretion [37]. Furthermore, IGF2 expression in liver has been reported to be regulated by GH [38]. Concordantly in our models, forced expression of hGH or hPRL increased IGF1 mRNA in Bel-7404 cells *in vitro* and increased serum hIGF1 in xenograft bearing hosts. In seeming contradiction, serum IGF1 levels have been reported to be decreased in patients with HCC [39] and the decreased serum IGF1 concentrations in HCV dependent HCC is apparently largely independent of liver function [40]. However, tumor expression of IGF1 mRNA in HCC was reported to be significantly increased compared with adjacent non-tumor tissue [41]. In contrast, IGF2 expression is normally suppressed in adult tissues and reactivated in a number of different neoplastic disorders including HCC [42]. hGH and hPRL stimulation of IGF1 or IGF2 in HCC could therefore potentially mediate some of the oncogenic effects of hGH and hPRL. In support of this notion, inhibition of the IGF1R with either antibodies [43] or kinase inhibitor [44] exerts antineoplastic effects in human HCC cell lines. Autocrine produced hGH and hPRL could presumably also exert IGF1 and IGF2 independent oncogenic effects in HCC as has been previously reported for mammary carcinoma cells [45].

We demonstrated herein that the oncogenic effects of autocrine hGH and hPRL in HCC cells were mediated by STAT3 concordant with the previous report in endometrial carcinoma [46]. Constitutively activated STAT3 is observed in the majority of HCC but not in normal liver nor adjacent non-tumor tissue [32]. The levels of pSTAT3-Y705 are also associated with histological grade and tumor microvessel density in HCC [32] and larger tumor size, VEGF and Ki67 expression, higher clinical stage and poor patient survival [47]. However, the molecular mechanisms that promote STAT3 activation in HCC are largely unknown. It has been proposed that STAT3 activation in cancer cells is often mediated by cytokines and/or growth factors synthesized within the tumor microenvironment [48]. Experiments herein demonstrated that hGH or hPRL act as tumor produced autocrine or paracrine growth factors that promote the activation of STAT3. Autocrine expression of hGH or hPRL also promoted activation of ERK1/2 which is a positive regulator of STAT3 (Supplementary Figure S6). Interestingly, autocrine expression of hGH or hPRL also increased the expression of TNF (Supplementary Table S3), a proinflammatory factor involved in STAT3 activation, liver inflammation and tumorigenesis [33], suggesting that STAT3 may be directly or indirectly activated by autocrine expression of hGH or hPRL. We did not observe STAT5 activation in the HCC cell lines with forced expression of hGH or hPRL and hence the effects of hGH and hPRL appear to be independent of STAT5. In this regard it should be noted that GH utilizes STAT5 as an enhancer of IGF-1 gene transcription and there are no STAT5 response elements in the IGF-1 promoter [49]. It should also be noted that STAT3 may promote IGF-1 gene transcription in response to GH albeit less efficiently than STAT5 [50]. This observation would indeed be consistent with our results herein where the increase in IGF-1 mRNA expression, while significant, is not large.

Recently, a report demonstrating that PRL contributed to the proliferation of liver cancer cells via JAK2 signaling was published [26]. In that report, the serum PRL level was observed to be significantly higher in HCC patients compared to normal controls. It was further reported that PRL promoted JAK2 and STAT3 phosphorylation and Cyclin D1 expression in HepG2 cells [26]. Whilst the organ source of increased PRL in HCC was not identified, this report confirmed our observation of PRL dependent activation of STAT3 in HCC cells [26]. More recently, a controversial study demonstrated that PRL can protect mice from HCC was reported [51]. From their findings, PRL interacted with short form PRLR to constrain tumor promoting liver inflammation. However, as the authors also observed, human HCC cell lines (including the HepG2 cell line used in this study) express the predominant long form of PRLR which activates different pathways compared with the short form PRLR [51]. Therefore, which PRLR isoform is predominant in human HCC clinical samples requires investigation.

Evidence from experimental models is emerging that functional antagonism of hGH or hPRL is indicated to inhibit progression of tumors such as meningioma, breast, colorectal, endometrial and prostate carcinoma [4, 52–57]. Previous studies have demonstrated that B2036, a hGH antagonist, decreased oncogenicity of endometrial carcinoma cells [4] and decreased proliferation of primary human mammary carcinoma cells *in vitro* [58]. Furthermore, Pegvisomant, the pegylated form of B2036 with FDA approval for the treatment of acromegaly, produced shrinkage of mammary carcinoma xenografts associated with reduced proliferation and increased apoptosis [54]. Similarly, hPRL-G129R is a specific hPRLR antagonist [34]. hPRL-G129R inhibits proliferation by induction of apoptosis in hPRLR positive breast cancer cell lines [34] and prevent early stages of prostate tumorigenesis [57]. Herein, we have therefore used the hGH-G120R mutant to demonstrate that combined inhibition of both autocrine hGH and hPRL decreased oncogenicity of human HCC cell lines. hGH-G120R inhibits hGH binding to either hGH or PRL receptors and also hPRL binding to the hPRL receptor [29]. As previously proposed for mammary and endometrial carcinoma, use of a single dual antagonist to hGH and hPRL may be a preferred approach to inhibit the oncogenic actions of these hormones in HCC as opposed to use of specific antagonists individually.

## MATERIALS AND METHODS

### Ethics statement

Investigations have been conducted in accordance with the ethical standards according to the Declaration of Helsinki and according to national and international guidelines and has been approved by the authors' institutional review board.

### Patients and specimens

Formalin-fixed and paraffin-embedded, HCC and non-neoplastic liver specimens (n=148) were obtained from the Department of Pathology of the First Affiliated Hospital of Anhui Medical University between 2004 and 2007. Fresh HCC and adjacent non-neoplastic liver tissues were immediately mixed with RNAlater and stored at −80°C freezer. The pathological tumor stage was defined according to the tumor-node-metastasis (TNM) classification of the International Union against Cancer (6^th^ Edition). The Edmondson grading system was used to define tumor differentiation [59]. Complete follow-up data were obtained on all HCC patients to determine overall survival (OS) and relapse-free survival (RFS). A protocol to use patient samples was approved by the Biomedical Ethics Committee of Anhui Medical University and a written informed consent was obtained from each patient.

### Tissue microarray (TMA) Construction, *In situ* hybridization (ISH) and Immunohistochemistry (IHC)

TMA constructions and ISH and IHC for hGH and hPRL were performed as previously described [8]. The diameter of each tissue core in the TMA was 1 mm and three to five representative were obtained from each case and inserted in a grid pattern into a recipient paraffin block. The evaluation of ISH and IHC staining was based on the combined expression pattern of all of the tissue cores from each patient sample. Stained sections were independently assessed for expression of hGH and hPRL with a light microscope by two pathologists without knowledge of the samples associated clinicopathologic information. The sections were scored on the basis of the percentage of cells with staining relative to the background and the staining intensity. Firstly, the extent of staining was scored as 0 (0%), 1 (1%-25%), 2 (26%-50%), 3 (51%-75%), and 4 (76%-100%) according to the percentage of the positive staining areas and staining intensity was scored as 0 (negative), 1 (weak), 2 (medium), and 3 (strong). The sum of the extent and intensity score was used as the staining score (0-7) for hGH and hPRL expression. Scores of 0-1, 2-3, 4-5 and 6-7 was designated as −, +, ++ and +++ respectively. Scores of – and + was designated as low expression and ++ and +++ was designated as high expression [60].

### Cell lines and reagent

Human cell lines HepG2 (hepatoblastoma cell line) and HCC cell line PLC/PRF/5 were purchased from ATCC (Rockville, MD, USA). LO2, Bel-7404, QGY-7701, QGY-7703 and SMMC-7721 cells were kindly provided by Dr. Lijian Hui (Institute of Biochemistry and Cell Biology, Chinese Academy of Sciences). All cells were maintained in DMEM (Gibco, Grand Island, NY, USA) medium plus 10% Fetal bovine serum (Hyclone, Beijing, China). STAT3 inhibitor cryptotanshinone was purchased from SigmaAldrich (St Louis, MO, USA).

### Constructs, plasmid and siRNA transfection

The plasmid pcDNA3-hGH was constructed as previously described [4]. The hPRL (Genebank accession number: NM_000948.5) cDNA was subcloned into pcDNA 3 plasmid. pcDNA3-G120R plasmid was generated using a QuickChange Site-Directed Mutagenesis Kit (Stratagene, La Jolla, CA, USA). Bel-7404 and HepG2 stable cell lines (pooled) were established by plasmids transfection by Lipofectamine 2000 (Invitrogen, Carlsbad, CA, USA) and then growing cell in G418 (800 μg/ml, SigmaAldrich) containing medium for two weeks. siRNAs targeting hGH (SI03053498 and SI03076311) and hPRL (SI00019019 and SI00019033) were purchased from Qiagen (Hilden, Germany). The four individual siRNAs were mixed equally for transfection by use of HiPerFect transfection reagent (Qiagen).

### Semi-quantitative RT-PCR and real-time quantitative PCR

Total RNA was extracted from cultured cells or fresh tissues with Trizol (Invitrogen). Semi-quantitative RT-PCR and real-time quantitative PCR were performed as previously described [4]. Oligonucleotide sequences are listed in Supplementary Table S6 and S7. Sequences of the other primers used are as described previously [4].

### *In vitro* oncogenicity assays

Total cell number, BrdU incorporation, measurement of apoptosis (TUNEL assay), soft agar colony formation and three-dimensional Matrigel growth were performed as previously described [4].

### Immunoblot

Immunoblot was performed as previously described [61] by using the following antibodies: hGH (anti-hGH-2, 1:10000, NIDDK, Bethesda, MD, USA); hPRL (anti-hPRL-IC-5, 1:10000, NIDDK); STAT3 (P30007, 1:1000, Abmart, Shanghai, China); phospho-STAT3 (Y705) (ab76315, 1:10000, Abcam, Cambridge, United Kingdom), Phospho-p44/42 MAPK (Thr202/Tyr204) (9101, 1:1000, Cell Signaling), p44/42 MAPK (4696, 1:1000, Cell Signaling) and GAPDH (M20028, 1:5000, Abmart).

### ELISA

Cells were grown to 90% confluence in six-well plates. The medium was then changed to serum-free medium for 48h. Quantification of hGH (ab100526, Abcam) and hPRL (DY682, R&D Systems, Minneapolis, MN, USA) in cell culture conditioned medium and mouse serum hIGF1 (ab100545, Abcam) levels were performed with commercially available ELISA kits as manufactures' instructions.

### Immunofluorescence

Immunostaining of hGH and hPRL were performed with a rabbit anti-hGH antibody (anti-hGH-2, 1:150, NIDDK, Bethesda, MD, USA) or rabbit anti-hPRL antibody (anti-hPRL-IC-5, 1:150, NIDDK).

### Xenograft

The mice were maintained in a pathogen-free barrier environment and closely monitored by animal facility staff. All mice work procedures were approved by University of Science and Technology (USTC) Ethics Committee for Animal Care and Use and were performed in accordance with the regulations of animal care of USTC and conformed to the legal mandates and national guidelines for the care and maintenance of laboratory animals. Bel-7404 stable cell lines (Two million) were s.c injected into the right and left flank of BALB/c-nu/nu mice (Slaccas, Shanghai, China). Each group contained six mice. Tumor volume calculation, BrdU and TUNEL immunostaning were performed as previously described [61].

### Statistical analysis

All statistical analyses were performed as previously described [8].

## SUPPLEMENTARY FIGURES AND TABLES


