# Supplementary material for: Human growth hormone and human prolactin function as autocrine/paracrine promoters of progression of hepatocellular carcinoma
Source: Oncotarget. 2016 Apr 18;7(20):29465–79. doi: 10.18632/oncotarget.8781 (PMC5045410; doi:10.18632/oncotarget.8781)
Supplement: Supplementary file 1 [file oncotarget-07-29465-s001.pdf]

## SUPPLEMENTARY FIGURES AND TABLES

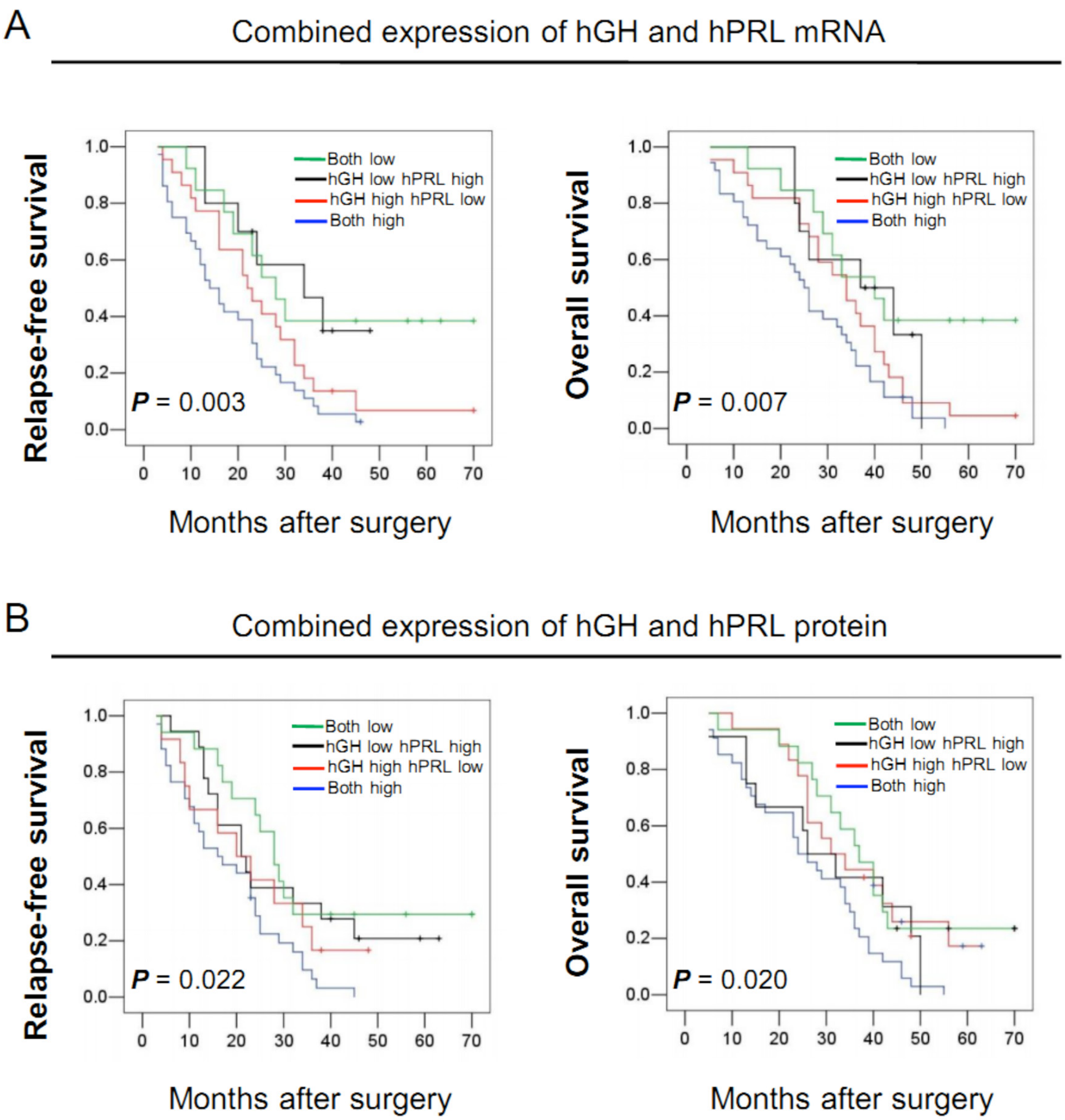


**Supplementary Figure S1: Kaplan-Meier analysis of the significance of hGH and hPRL expression on RFS and OS of male patients with HCC. A.** The relationship of combined expression of hGH and hPRL mRNA and RFS to OS of male patients with HCC. **B.** The relationship of combined expression of hGH and hPRL protein and RFS to OS of male patients with HCC.

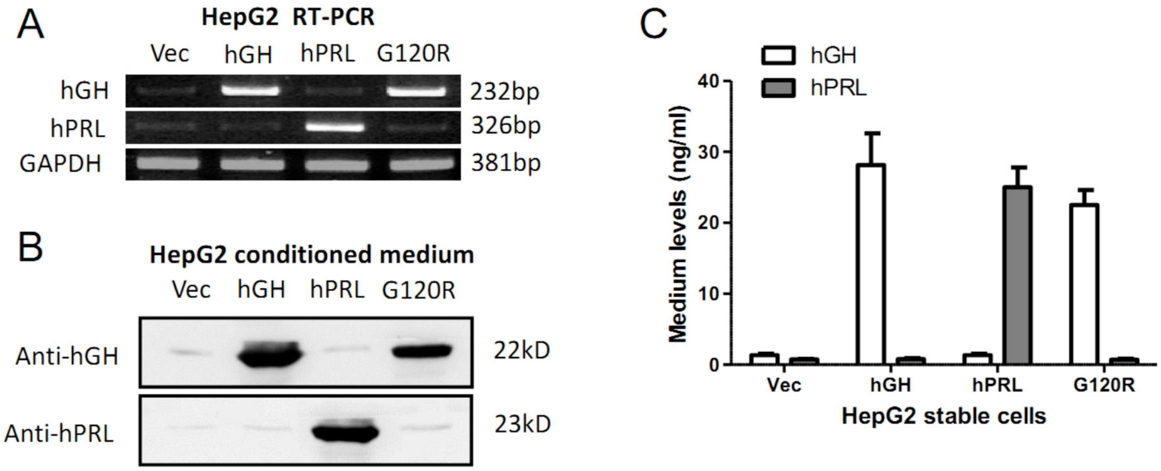


**Supplementary Figure S2: Forced expression of hGH, hPRL and G120R in HepG2 cells. A.** RT-PCR analyses of forced expression of hGH, hPRL and G120R in HepG2 cells. **B.** Immunoblot analyses of forced expression and secretion of hGH, hPRL and G120R in HepG2 cells. **C.** ELISA detection of forced expression and secretion of hGH, hPRL and G120R in HepG2 cells. Mean +/− SD.

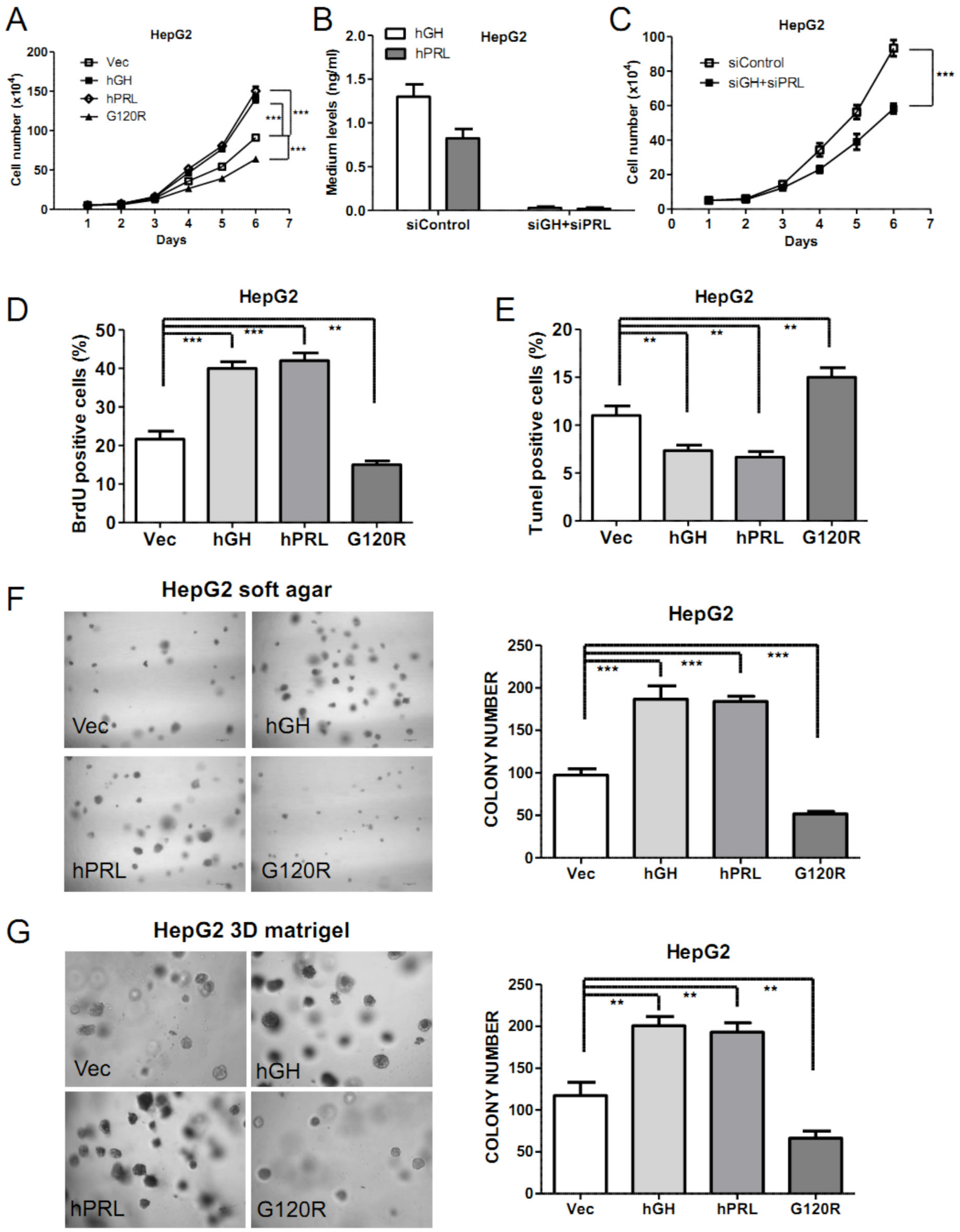


**Supplementary Figure S3: Autocrine expression of hGH or hPRL promotes oncogenicity of HepG2 cells *in vitro*. A.** Growth of HepG2 stable cell lines was assessed by a total cell number assay in complete medium. **B.** ELISA detection of hGH and hPRL levels in medium of HepG2 cells with combined transfection of hGH and hPRL siRNAs. Cells were incubated in serum free DMEM for 48 hours prior analysis. **C.** Growth of HepG2 cells with combined transfection of hGH and hPRL siRNAs by a total cell number assay in complete medium. **D.** Effect of autocrine expression of hGH or hPRL on cell cycle progression as assessed by nuclear BrdU incorporation in complete medium. **E.** Effect of autocrine expression of hGH or hRPL on apoptosis induced by serum withdrawal over 48 h as evaluated by TUNEL assay. **F.** Soft agar colony formation of HepG2 stable cell lines. **G.** 3D Matrigel growth by HepG2 stable cell lines. ** $p<0.01$, *** $p<0.001$. Mean +/− SD.

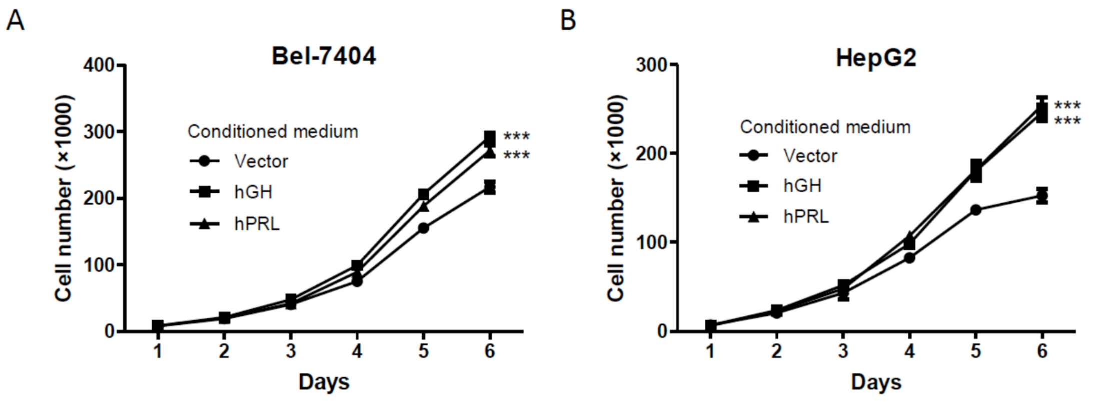


**Supplementary Figure S4: Conditioned medium from HCC cells with either forced expression of hGH or hPRL promoted proliferation of the respective parental cell line.** **A.** Growth of Bel-7404 cells cultured with conditioned medium collected from Bel-7404 stably expressing Vector, hGH or hPRL cells was assessed by a total cell number assay. **B.** Growth of HepG2 cells cultured with conditioned medium collected from HepG2 stably expressing Vector, hGH or hPRL cells was assessed by a total cell number assay. *** p<0.001. Mean +/− SD.

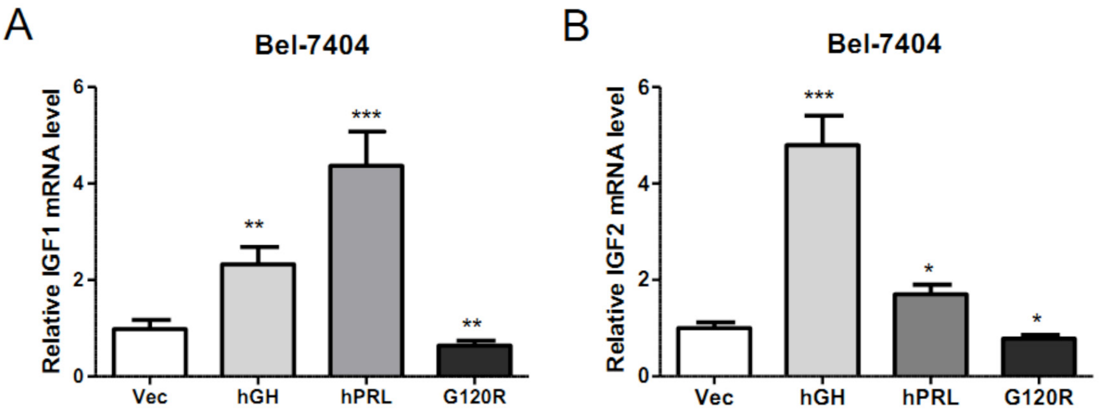


**Supplementary Figure S5: Autocrine expression of hGH or hPRL modulate IGF1 and IGF2 mRNA levels in Bel-7404 cells.** **A.** Relative IGF1 mRNA levels in Bel-7404 stable cell lines determined by qPCR. **B.** Relative IGF2 mRNA levels in Bel-7404 stable cell lines determined by qPCR. * p<0.05, ** p<0.01, *** p<0.001. Mean +/− SD.

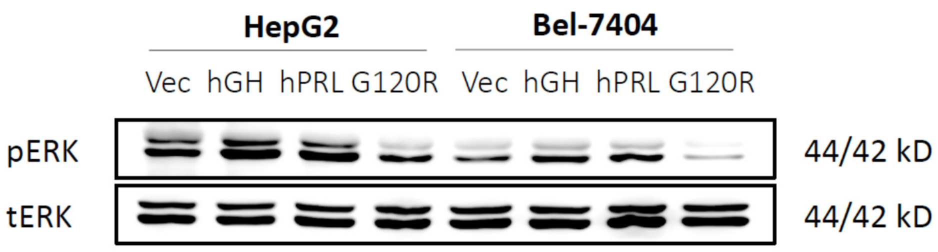


**Supplementary Figure S6: Autocrine expression of hGH or hPRL in HepG2 and Bel-7404 cells increase ERK1 and ERK2 activation.** Total cell lysates of HepG2 and Bel-7404 stable cell lines were subjected to western blot analysis to detect pERK1/2 expression.

**Supplementary Table S1: Expression of hGH or hPRL mRNA and hGH, hPRL protein in hepatocelluar carcinoma and adjacent non-neoplastic hepatic tissue**

| | | **hGH mRNA expression n (%)** | | | | **hGH protein expression n (%)** | | | |
|---|---|---|---|---|---|---|---|---|---|
| Group | *n* | − | + | ++ | +++ | − | + | ++ | +++ |
| Non-neoplastic | 132 | 14(10) | 46(34.8) | 42(31.8) | 30(22.7) | 5(3.8) | 61(46.2) | 54(40.9) | 12(9.1) |
| Carcinoma | 148 | 14(9.5) | 31(20.9) | 33(22.3) | 70(47.3) | 5(3.4) | 50(33.8) | 62(41.9) | 31(20.9) |
| *P* | | <0.001 | | | | 0.027 | | | |
| | | hPRL mRNA expression n (%) | | | | hPRL protein expression n (%) | | | |
| Group | *n* | − | + | ++ | +++ | − | + | ++ | +++ |
| Non-neoplastic | 128 | 39(30.5) | 46(35.9) | 31(24.2) | 12(9.4) | 44(34.4) | 74(57.8) | 9(7) | 1(0.8) |
| Carcinoma | 145 | 23(15.9) | 46(31.7) | 38(26.2) | 38(26.2) | 19(13.1) | 56(38.6) | 32(22.1) | 38(26.2) |
| *P* | | 0.001 | | | | <0.001 | | | |

**Supplementary Table S2: Association of tumor hGH or hPRL mRNA and tumor hGH or hPRL protein expression with clinicopathological parameters of patients with hepatocellular carcinoma**

| Parameter | hGH high expression *n* (%) | | | | | | hPRL high expression *n* (%) | | | | | |
|---|---|---|---|---|---|---|---|---|---|---|---|---|
| | *n* | mRNA | *P* | *n* | protein | *P* | *n* | mRNA | *P* | *n* | protein | *P* |
| **Age (years)** | | | | | | | | | | | | |
| ≤ 55 | 92 | 65 (70.7) | 0.72 | 92 | 59 (64.1) | 0.677 | 89 | 45 (50.6) | 0.573 | 89 | 43 (48.3) | 0.991 |
| > 55 | 56 | 38 (67.9) | | 56 | 34 (60.7) | | 56 | 31 (55.4) | | 56 | 27 (48.2) | |
| **Gender** | | | | | | | | | | | | |
| Male | 124 | 90 (72.6) | 0.073 | 124 | 83 (66.9) | **0.019** | 122 | 71 (58.2) | **0.001** | 122 | 63 (51.6) | 0.062 |
| Female | 24 | 13 (54.2) | | 24 | 10 (41.7) | | 23 | 5 (21.7) | | 23 | 7 (30.4) | |
| **Cirrhosis** | | | | | | | | | | | | |
| Yes | 134 | 92 (68.7) | 0.443 | 134 | 86 (64.2) | 0.296 | 131 | 69 (52.7) | 0.849 | 131 | 61 (46.6) | 0.207 |
| No | 14 | 11 (78.6) | | 14 | 7 (50.0) | | 14 | 7 (50.0) | | 14 | 9 (64.3) | |
| **HBsAg** | | | | | | | | | | | | |
| Yes | 118 | 81 (68.6) | 0.618 | 118 | 78 (66.1) | 0.103 | 115 | 62 (53.9) | 0.479 | 115 | 56 (48.7) | 0.843 |
| No | 30 | 22 (73.3) | | 30 | 15 (50.0) | | 30 | 14 (46.7) | | 30 | 14 (46.7) | |
| **Tumor size (cm)** | | | | | | | | | | | | |
| < 5 | 43 | 25 (58.1) | **0.042** | 43 | 30 (69.8) | 0.264 | 41 | 17 (41.5) | 0.097 | 41 | 16 (39.0) | 0.162 |
| ≥ 5 | 105 | 78 (74.3) | | 105 | 63 (60.0) | | 104 | 59 (56.7) | | 104 | 54 (51.9) | |
| **Grade** | | | | | | | | | | | | |
| I | 5 | 1 (20.0) | **0.045** | 5 | 2(40.0) | 0.129 | 5 | 1 (20.0) | 0.332 | 5 | 1 (20.0) | 0.253 |
| II | 138 | 98 (71.0) | | 139 | 86 (61.9) | | 136 | 73 (53.7) | | 136 | 66 (48.5) | |
| III | 5 | 4 (80.0) | | 5 | 5 (100.0) | | 4 | 2 (50.0) | | 4 | 3 (75.0) | |
| **Stage** | | | | | | | | | | | | |
| I | 3 | 1 (33.3) | 0.553 | 3 | 1 (33.3) | 0.669 | 3 | 2 (66.7) | 0.628 | 3 | 1 (33.3) | 0.591 |
| II | 111 | 79 (71.2) | | 111 | 72 (64.9) | | 109 | 59 (54.1) | | 109 | 56 (51.4) | |
| III | 10 | 7 (70.0) | | 10 | 6 (60.0) | | 9 | 3 (33.3) | | 9 | 3 (33.3) | |
| IV | 24 | 16 (66.7) | | 24 | 14 (58.3) | | 24 | 12 (50.0) | | 24 | 10 (41.7) | |

**Supplementary Table S3: Multivariate analysis of tumor hGH or hPRL mRNA and hGH or hPRL protein expression with five year relapse free (RFS) and overall survival (OS) in patients with hepatocellular carcinoma**

| | RFS -Odds ratio (95% CI) | | | | OS-Odds ratio (95% CI) | | | |
|---|---|---|---|---|---|---|---|---|
| | mRNA | *P* | protein | *P* | mRNA | *P* | protein | *P* |
| hGH low/hGH high | 1.336 (1.047-1.706) | **0.02** | 1.154 (0.925-1.438) | 0.204 | 1.299 (1.023-1.649) | **0.032** | 1.121 (0.904-1.391) | 0.299 |
| hPRL low/hPRL high | 1.196 (0.962-1.487) | 0.106 | 1.761 (1.131-2.742) | **0.012** | 1.241 (0.999-1.541) | 0.051 | 1.676 (1.084-2.59) | **0.02** |
| hGH low hPRL low /hGH high | 1.23 (1.006-1.503) | **0.044** | 1.148 (0.959-1.374) | 0.133 | 1.263 (1.033-1.544) | **0.023** | 1.103 (0.928-1.311) | 0.103 |
| hGH low hPRL low /hPRL high | 1.208 (0.98-1.488) | 0.077 | 1.206 (1.005-1.448) | **0.044** | 1.264 (1.023-1.56) | **0.019** | 1.148 (0.963-1.367) | 0.123 |
| hGH low hPRL low/hGH high hPRL high | 1.306 (1.053-1.619) | **0.015** | 1.27 (1.047-1.54) | **0.015** | 1.327 (1.069-1.646) | **0.01** | 1.216 (1.01-1.464) | **0.038** |

**Supplementary Table S4: Association of tumor hGH or hPRL mRNA and hGH or hPRL protein expression with five year relapse free (RFS) and overall survival (OS) in male and female patients with hepatocellular carcinoma**

| | | Hepatocellular carcinoma | | | | | | | |
|---|---|---|---|---|---|---|---|---|---|
| | | RFS (%) | | | | | | OS (%) | |
| | | mRNA | *P* | protein | *P* | mRNA | *P* | protein | *P* |
| hGH low/ hGH high | male | 37.5/ 6.7 | **0.003** | 23.3/ 11.1 | 0.107 | 33.3/ 5.0 | **0.008** | 20.3/ 9.3 | 0.175 |
| | female | 12.5/ 25.0 | 0.478 | 18.2/20.0 | 0.917 | 12.5/ 12.5 | 0.726 | 9.1/ 20.0 | 0.866 |
| hPRL low/ hPRL high | male | 19.4/ 10.9 | 0.09 | 25.0/ 6.5 | **0.006** | 16.7/ 8.7 | **0.036** | 22.2/4.3 | **0.006** |
| | female | 21.4/ 0 | 0.497 | 27.3/ 0 | 0.758 | 14.3/ 0 | 0.866 | 18.2/ 0 | 0.667 |

**Supplementary Table S5: qPCR Analysis of Modulation of Gene Expression by Forced Expression of hGH, hPRL or G120R in Bel-7404 cell**

| | hGH/vector | | hPRL/vector | | hGH-120R/vector | |
|---|---|---|---|---|---|---|
| Genes | Ratio | p-value | Ratio | p-value | Ratio | p-value |
| CCND1 | 5.95 | 4.96E-04 | 9.66 | 1.85E-04 | 0.01 | 3.88E-05 |
| ATM | 1.37 | 1.64E-02 | 20.87 | 3.44E-05 | 0.21 | 1.00E-04 |
| BRCA1 | 1.16 | 2.96E-01 | 2.29 | 1.31E-03 | 1.42 | 5.94E-03 |
| CCNE1 | 2.41 | 5.12E-03 | 3.01 | 3.08E-03 | 0.01 | 2.82E-04 |
| CDC25A | 4.56 | 3.01E-04 | 2.43 | 8.43E-04 | 0.01 | 1.97E-03 |
| CDK2 | 2.29 | 3.47E-03 | 0.77 | 1.46E-03 | 0.41 | 1.51E-02 |
| CDK4 | 3.38 | 1.50E-04 | 2.98 | 8.39E-05 | 0.01 | 1.25E-05 |
| CDKN1A | 2.22 | 5.10E-03 | 0.32 | 1.11E-03 | 0.19 | 7.64E-04 |
| CDKN2A | 3.14 | 1.17E-03 | 3.29 | 1.45E-04 | 0.6 | 3.94E-04 |

(*Continued*)

| | hGH/vector | | hPRL/vector | | hGH-120R/vector | |
|---|---|---|---|---|---|---|
| Genes | Ratio | p-value | Ratio | p-value | Ratio | p-value |
| CHEK2 | 1.36 | 7.56E-03 | 0.96 | 3.78E-03 | 0.56 | 4.53E-03 |
| E2F1 | 1.24 | 3.87E-01 | 0.21 | 6.74E-04 | 0.19 | 6.67E-04 |
| MDM2 | 2.75 | 2.18E-03 | 19.07 | 4.52E-05 | 13989.52 | 3.36E-06 |
| RB1 | 0.96 | 1.34E-04 | 0.02 | 8.54E-06 | 0.01 | 5.15E-05 |
| S100A4 | 0.47 | 1.30E-03 | 0.06 | 1.01E-04 | 0.05 | 1.04E-04 |
| TP53 | 0.13 | 5.04E-04 | 0.01 | 4.47E-05 | 2.82 | 1.59E-03 |
| CDKN1B | 0.26 | 1.13E-04 | 0.86 | 4.43E-03 | 5.11 | 1.24E-04 |
| APAF1 | 1.16 | 1.94E-02 | 0.14 | 1.18E-05 | 0.13 | 3.47E-04 |
| BCLAF1 | 0.26 | 4.05E-04 | 0.18 | 1.17E-04 | 2.22 | 5.08E-04 |
| BAK1 | 0.31 | 3.08E-05 | 17.3 | 1.37E-05 | 10.39 | 3.17E-06 |
| BAD | 0.22 | 1.35E-03 | 0.14 | 2.50E-04 | 19.91 | 1.32E-04 |
| BAX | 12.03 | 5.22E-04 | 0.01 | 7.96E-05 | 0.04 | 5.15E-04 |
| BCL2 | 21.2 | 1.18E-04 | 50.53 | 6.01E-05 | 0.15 | 1.36E-04 |
| CFLAR | 0.47 | 5.90E-04 | 4.44 | 2.82E-04 | 3.56 | 1.38E-03 |
| CASP7 | 0.28 | 3.40E-03 | 0.01 | 1.67E-03 | 5.93 | 1.19E-03 |
| GZMA | 22.15 | 5.14E-05 | 177.89 | 2.14E-05 | 0.08 | 2.45E-04 |
| HTATIP2 | 0.04 | 8.71E-04 | 0.05 | 6.25E-05 | 13.37 | 8.97E-05 |
| TERT | 18.84 | 1.08E-03 | 22.53 | 1.48E-03 | 0.08 | 1.11E-03 |
| TNFRSF1A | 5.32 | 1.60E-03 | 0.43 | 4.11E-03 | 0.45 | 7.74E-03 |
| TNFRSF10B | 1.35 | 9.03E-03 | 0.01 | 6.53E-07 | 0.01 | 8.64E-07 |
| TNFRSF25 | 12.58 | 3.26E-03 | 181.64 | 3.55E-05 | 0.22 | 6.47E-04 |
| AKT1 | 1.12 | 2.41E-01 | 18.29 | 1.62E-04 | 0.06 | 1.10E-02 |
| ERBB2 | 0.98 | 5.04E-03 | 0.05 | 1.47E-05 | 0.8 | 2.98E-02 |
| ETS2 | 1.7 | 3.71E-04 | 0.86 | 4.20E-01 | 0.01 | 2.24E-06 |
| FOS | 1.11 | 3.49E-03 | 45.22 | 3.09E-07 | 1.16 | 1.43E-01 |
| JUN | 1 | 2.54E-02 | 0.01 | 2.43E-05 | 0.01 | 1.49E-05 |
| MAP2K1 | 2.09 | 4.43E-03 | 2.46 | 1.22E-03 | 0.01 | 1.40E-04 |
| MYC | 0.86 | 8.30E-03 | 1.54 | 1.31E-02 | 0.01 | 7.04E-05 |
| NFKB1 | 0.73 | 1.17E-03 | 2.52 | 8.81E-03 | 1.82 | 1.25E-03 |
| PIK3R1 | 2.7 | 1.50E-02 | 0.05 | 1.11E-03 | 0.06 | 1.78E-03 |
| RAF1 | 1.12 | 1.14E-01 | 0.65 | 5.54E-04 | 0.38 | 2.22E-04 |
| SNCG | 4.55 | 1.47E-02 | 54.47 | 4.57E-04 | 0.36 | 7.98E-03 |
| ANGPT1 | 2.15 | 2.26E-04 | 12.47 | 1.44E-04 | 0.01 | 7.81E-04 |
| ANGPT2 | 0.78 | 5.79E-04 | 14 | 4.71E-04 | 0.15 | 8.87E-04 |
| VEGF | 2.16 | 2.08E-03 | 3.23 | 4.93E-04 | 0.1 | 9.47E-05 |
| COL18A1 | 0.78 | 3.84E-02 | 1.36 | 1.07E-01 | 0.63 | 2.21E-02 |

(*Continued*)

| | hGH/vector | | hPRL/vector | | hGH-120R/vector | |
|---|---|---|---|---|---|---|
| **Genes** | **Ratio** | **p-value** | **Ratio** | **p-value** | **Ratio** | **p-value** |
| IL8 | 0.73 | 3.41E-03 | 0.2 | 3.27E-04 | 0.21 | 1.59E-04 |
| TEK | 0.63 | 1.04E-03 | 61.29 | 4.44E-05 | 45.56 | 4.88E-05 |
| TGFB1 | 1.09 | 5.12E-02 | 0.05 | 2.34E-05 | 0.03 | 2.10E-05 |
| TGBS1 | 1.04 | 2.45E-02 | 0.94 | 4.96E-03 | 0.57 | 2.32E-03 |
| TNF | 8.87 | 5.47E-05 | 1580.73 | 4.98E-06 | 0.13 | 1.01E-03 |

**Supplementary Table S6: Sequences of oligonucleotide primers used for semiquantitative RT-PCR**

| Genes | Abbreviation | Primer Sequence (5'-3') | | Amplicon Size (bp) |
|---|---|---|---|---|
| Glyceraldehyde-3-phosphate dehydrogenase | GAPDH | Forward | TCCCATCACCATCTTCCAGG | 381 |
| | | Reverse | CCATCACGCCACAGTTTCC | |
| Growth hormone 1 | GH1 | Forward | GGCGCCTCTGACAGCAACGTCT | 232 |
| | | Reverse | GCACGATGCGCAGGAATGTCTC | |
| Growth hormone receptor | GHR | Forward | CAGCAGCCCAGTGTTAT | 247 |
| | | Reverse | CTTGGCAGAGTGAGACC | |
| Prolactin | PRL | Forward | GTCCCACTACATCCATAACCTC | 326 |
| | | Reverse | CTATCAGCTCCATGCCCTCT | |
| Prolactin receptor | PRLR | Forward | TACTGAGTGCCTTGGGATGC | 135 |
| | | Reverse | CTTGACTTGGGTGTTCTTTTGA | |

**Supplementary Table S7: Sequences of oligonucleotide primers used for quantitative RT-PCR**

| Genes | Abbreviation | Primer Sequence (5'-3') | | Amplicon Size (bp) |
|---|---|---|---|---|
| Glyceraldehyde-3-phosphate dehydrogenase | GAPDH | Forward | TGCACCACCAACTGCTTAGC | 84 |
| | | Reverse | GGCATGGACTGTGGTCATGAG | |
| Growth hormone 1 | GH1 | Forward | GGCGCCTCTGACAGCAACGTCT | 232 |
| | | Reverse | GCACGATGCGCAGGAATGTCTC | |
| Insulin-like growth factor 1 (somatomedin C) | IGF1 | Forward | TGGTGGATGCTCTTCAGTTC | 264 |
| | | Reverse | CACTCCCTCTACTTGCGTTCT | |
| Insulin-like growth factor 2 (somatomedin A) | IGF2 | Forward | CGTGGCATCGTTGAGGAGT | 301 |
| | | Reverse | GGGTGGGTAGAGCAATCAGG | |
| Prolactin | PRL | Forward | GTCCCACTACATCCATAACCTC | 326 |
| | | Reverse | CTATCAGCTCCATGCCCTCT | |
